# Latent Adrenal Insufficiency: From Concept to Diagnosis

**DOI:** 10.3389/fendo.2021.720769

**Published:** 2021-08-27

**Authors:** Nada Younes, Isabelle Bourdeau, Andre Lacroix

**Affiliations:** Division of Endocrinology, Department of Medicine and Research Center, Centre Hospitalier de l’Université de Montréal (CHUM), Montréal, QC, Canada

**Keywords:** cortisol, aldosterone, ACTH, renin, autoimmunity, hypocortisolism, adrenal insufficiency

## Abstract

Primary adrenal insufficiency (PAI) is a rare disease and potentially fatal if unrecognized. It is characterized by destruction of the adrenal cortex, most frequently of autoimmune origin, resulting in glucocorticoid, mineralocorticoid, and adrenal androgen deficiencies. Initial signs and symptoms can be nonspecific, contributing to late diagnosis. Loss of zona glomerulosa function may precede zona fasciculata and reticularis deficiencies. Patients present with hallmark manifestations including fatigue, weight loss, abdominal pain, melanoderma, hypotension, salt craving, hyponatremia, hyperkalemia, or acute adrenal crisis. Diagnosis is established by unequivocally low morning serum cortisol/aldosterone and elevated ACTH and renin concentrations. A standard dose (250 µg) Cosyntropin stimulation test may be needed to confirm adrenal insufficiency (AI) in partial deficiencies. Glucocorticoid and mineralocorticoid substitution is the hallmark of treatment, alongside patient education regarding dose adjustments in periods of stress and prevention of acute adrenal crisis. Recent studies identified partial residual adrenocortical function in patients with AI and rare cases have recuperated normal hormonal function. Modulating therapies using rituximab or ACTH injections are in early stages of investigation hoping it could maintain glucocorticoid residual function and delay complete destruction of adrenal cortex.

## Definition and Epidemiology

The adrenal cortex produces glucocorticoids, mineralocorticoids and androgens, under the influence of adrenocorticotropic hormone (ACTH) and the renin-angiotensin system (1, 2). In the event of adrenal cortex destruction, primary adrenal insufficiency (PAI) develops and is characterized by reduced serum concentrations of all three hormones: cortisol, aldosterone and adrenal androgens (3). However, since intra-adrenal cortisol is required for epinephrine production by the adrenal medulla, PAI is often associated with decreased phenylethanolamine N-methyltransferase (PNMT) activity, resulting in adrenomedullary dysfunction (1, 4). Central adrenal insufficiency (AI), encompasses both secondary and tertiary AI caused by low ACTH and low corticotropin releasing hormone (CRH), respectively (5). However, since aldosterone production is mainly controlled by renin, angiotensin II and potassium (1, 6), it is not affected in central AI.

PAI was first described by Thomas Addison in 1855 in a case series of 11 patients and therefore it is often called Addison’s disease (7). Since then, prevalence has been on the rise especially in Europe, reaching 117/million in central Italy in the late 1990s (8) and 144/million in Norway in 2007 (9). More recently, an even higher prevalence was documented in a 2016 Icelandic nationwide study of patients over 18 years of age: 221/million population (10). Furthermore, an annual average increase in the prevalence of PAI of 1.8% per year was reported in Germany from 2008 to 2012 (11). The annual incidence of PAI is estimated to be around 0.44-0.62 per 100,000 (9, 12). PAI is more frequently found in women than men (11, 13) with a M:F ratio of 1: 3.5 (14). Age of onset is typically around 30-50 years old (1, 9, 10, 14). Autoimmune destruction of adrenal cortex has surpassed tuberculosis as the most common cause of PAI, in particular in high income countries (1, 15). Comorbid autoimmune disorders can often be found in patients with autoimmune AI (AAI), reported to be 46.5 and 66%, respectively in the German (11) and Norwegian studies (9). Most common comorbid conditions were thyroid disease, type 1 diabetes, vitiligo, vitamin B12 deficiency and primary ovarian insufficiency (POI) (9–11). When occurring with other autoimmune disorders, AAI may be part of an autoimmune polyglandular syndrome (APS). APS type 1 or autoimmune polyendocrinopathy–candidiasis–ectodermal dystrophy (APECED) is a rare monogenic disease, in which the autoimmune regulator gene (*AIRE*) is mutated and at least 2 of the following three occur: chronic mucocutaneous candidiasis, hypoparathyroidism and PAI (16). Generally, its transmission is autosomal recessive but may be dominant (16). Other conditions may include POI, enteropathy and rarely lymphomas (6, 16). More commonly found is APS type 2, a polygenic condition in which genes encoding cytotoxic T-lymphocyte-associated protein 4 (CTLA-4), protein tyrosine phosphatase, nonreceptor type 22 (PTPN22), the transcriptional regulator protein BACH2, and the CD25–interleukin-2 receptor have been implicated (16), along with associations to certain haplotypes including DR3-DQ2, DR4-DQ8, DRB1-0301, and DRB1-0404 (15, 17). It commonly associates with type 1 diabetes and autoimmune thyroid disease (16). When AAI is confirmed alongside other autoimmune diseases, excluding the cardinal ones needed for diagnosis of APS types 1 and 2, APS type 4 is diagnosed (5).

## Signs and Symptoms of AI- Natural History of Autoimmune PAI-Mortality

Onset of AI can be insidious, with many of the symptoms and signs being nonspecific and often leading to a delay in diagnosis (1). In a retrospective study including 216 patients with AI (18), more than half of patients consulted at least 3 physicians and were falsely diagnosed with either gastrointestinal or psychiatric disorders most frequently. A 5 year delay in diagnosis was reported in as much as 20% of patients (18). Symptoms of cortisol deficiency are common to both primary and central AI. These include loss of appetite, weight loss, lethargy, gastrointestinal symptoms such as nausea, abdominal pain and vomiting (1, 6).

Because aldosterone deficiency exists in PAI but not in central AI, symptoms and signs of mineralocorticoid deficiency including, dizziness and orthostatic hypotension, salt craving, hyponatremia, hyperkalemia and hyperchloremic acidosis, can be found (1, 19). Orthostatic hypotension may also be secondary to cortisol deficiency *via* reduced expression of catecholamine receptors on blood vessels and therefore may be found in central AI, albeit less pronounced than in PAI (1, 20). Although hyponatremia can be present in both primary and central AI, the underlying mechanisms differ. In PAI, hyponatremia is secondary to aldosterone deficiency resulting in renal salt wasting and hypovolemia with an increased risk of dehydration and acute kidney injury (19, 21). In central AI, it is a result of increased vasopressin secondary to cortisol deficiency, which in turn results in water retention and euvolemic hyponatremia (22). Other possible laboratory abnormalities are hypercalcemia, mild normocytic anemia, lymphocytosis, eosinophilia and hypoglycemia which occurred more frequently in children (1, 6). There is also a significant decrease in natural killer cells cytotoxicity in patients with PAI, compromising innate immunity, hence contributing to increased viral infections (23). Androgen deficiency may result in low libido or reduced energy as well as thinning of axillary and pubic hair in post-menopausal women (1, 3). In pregnant women with unrecognized AI, establishing the diagnosis based on signs and symptoms is challenging, because cortisol deficiency symptoms may also be seen as part of a normal pregnancy. While only a few cases were diagnosed during pregnancy, the high maternal and fetal risks associated with unrecognized AI should prompt physicians to suspect the diagnosis in women with symptoms persisting into the second trimester or occurring secondary to illness or labor (24).

A particularly distinctive feature of PAI is melanoderma which can be explained by elevated ACTH due to loss of negative feedback control usually exerted by cortisol on corticotropic cells in anterior pituitary gland (1). Elevated plasma ACTH activates melanocortin 1 receptors (M1R), resulting in excessive pigmentation, especially on areas exposed to sun and friction such as face, neck, knuckles, creases in the hand, elbows, areola of the nipple, scrotum, labia and newly acquired scars (6, 19). Mucosal pigmentation can also be noted and clinicians should look for brown patchy discoloration on lips, palate and gingiva (19).

**Figure 1:**
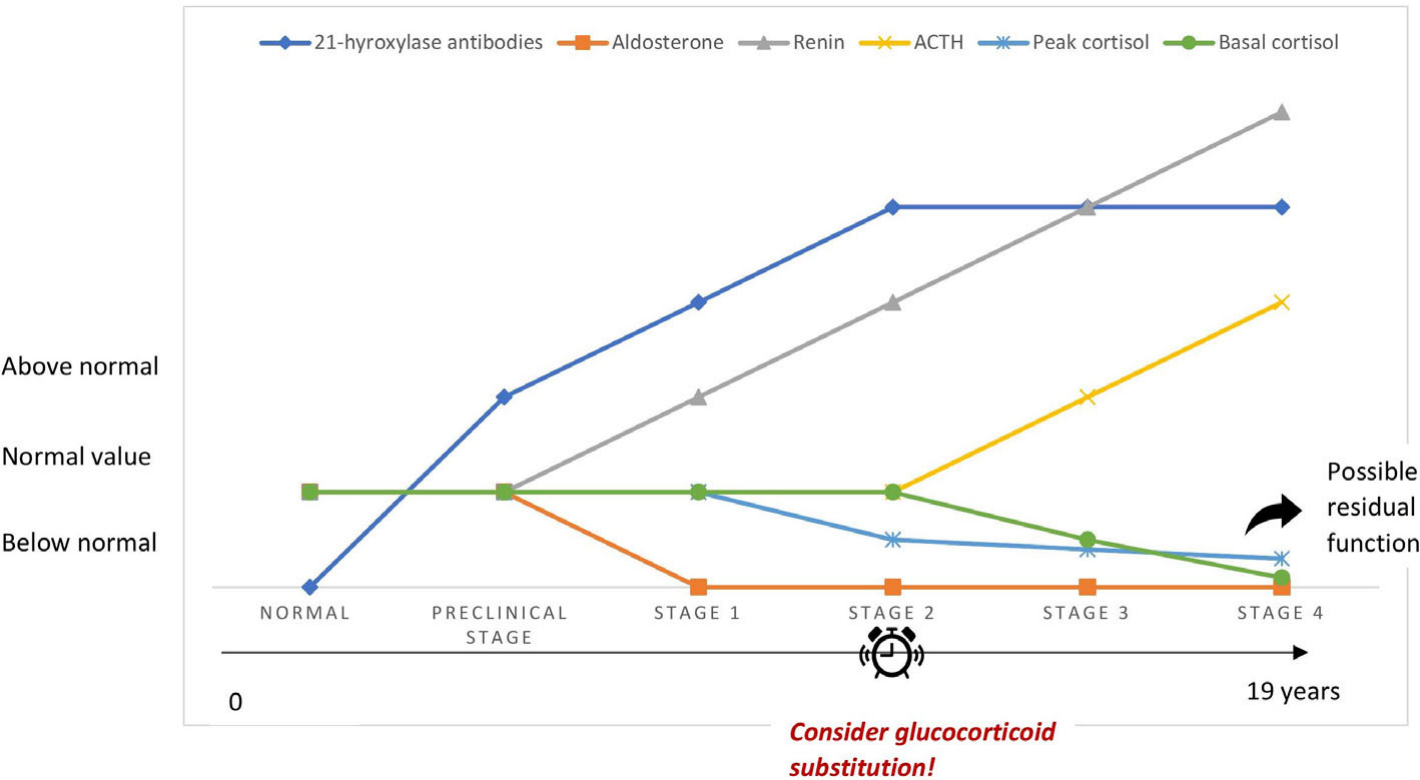
Simplified schematic depiction of the 4 stages of autoimmune adrenocortical destruction.

PAI may be underrecognized because of the nonspecific symptoms of cortisol deficiency, especially in its early stages. This is often called latent AI and should be suspected in the presence of unexplained health complaints related to stress, such as gastrointestinal symptoms, fatigue and weight loss, in particular in patients with a history of autoimmune disease (25, 26). Betterle et al. suggested that autoimmune adrenocortical destruction goes through 4 stages (27) (**Figure 1**). The very first stage of the disease is marked by aldosterone deficiency and elevated renin concentrations suggesting that zona glomerulosa may be more prone to autoimmune destruction than zona fasciculata (27–29). The latter may be initially spared owing to the high local concentrations of cortisol responsible for suppressing antigen presentation to immune cells and procuring local anti-inflammatory properties (30). The second stage is characterized by an impaired cortisol response to synthetic ACTH administered intravenously, while in third and fourth stages, ACTH increases and serum cortisol drops, respectively (27). Early stages of AAI are difficult to diagnose because of the asymptomatic or pauci-symptomatic state since adrenal function is maintained by high plasma ACTH/renin (31). Stages 1 and 2 may be reversible in sporadic AAI. This was not true in APS types 1 and 2/4, where respectively stage 1 and stage 2 were defined as the point of no return with a cumulative risk of 100% (28). Progression of AAI occurred in most patients within 11 years, while no progression was seen after 19 years of follow up in a large cohort of 143 antibody positive patients. Progression was higher in males, patients who had APS type 1 and impaired adrenal function (28). However, antibody titers did not affect risk of progression, probably because AAI is mediated by cellular immunity and antibodies are in fact markers of the disease (27, 28). In 100 asymptomatic antibody positive patients followed up for 20 years, impairment of adrenal function occurred at an annual rate of 4.9% (29). Treatment with glucocorticoids in stressful conditions such as a major surgery or illness may be considered for patients at stages 1 and 2 and close monitoring of patients with higher risk of progression to clinical disease is warranted (children, APS type 1, high antibody titers and already at stage 1) (29, 32).

Because of the insidious course of the disease, patients may not be diagnosed until they present with adrenal crisis. Adrenal crisis is an acute, life-threatening condition more frequently seen in PAI than central AI (33). The frequency of adrenal crisis varied from 4.4 to 17/100 patient-years (11, 34, 35), depending on the definition used for adrenal crises and the characteristics of patients included in each of the studies. A prospective study by Hahner et al., evaluated the incidence of adrenal crisis in 423 patients followed up for 2 years. It was found to be persistently high even in adequately educated patients, reaching 8.3 crises/100 patient-years with a high mortality, estimated at 0.5 adrenal crisis related deaths/100 patient-years (36). A much lower incidence was reported in Switzerland despite insufficient patient education regarding their disease (37). Recently, an analysis of the biggest cohort described to date, including 2694 patients from the European Adrenal Insufficiency Registry, revealed an adrenal crisis incidence of 6.53/100 patient-years, with more than one episode per year in 16% of patients with PAI (38). In the absence of a universal definition of adrenal crisis, its recognition can prove to be difficult. The most used definition is an unstable hemodynamic state (absolute or relative hypovolemia/shock) recovering within 1 to 2 hours following parenteral glucocorticoid administration (4, 33). Although hyponatremia and hyperkalemia are very common features in adrenal crisis, clinicians should beware of patients with multiple episodes of vomiting and severe dehydration because they can present with normal levels of serum potassium and sodium (6). Adrenal crisis can be triggered by numerous precipitating events, namely gastrointestinal infections, fever, emotional stress, major surgery, pregnancy, strenuous physical activity and noncompliance (34–37, 39). In 7-14% of adrenal crises, no precipitating event could be identified (34, 36). Patients at higher risk of adrenal crisis were especially those who have a history of a previous adrenal crisis (threefold increase) (36), but also patients that are female (35), older (40) and have diabetes insipidus (35), type 1 diabetes mellitus (41) and cardiac, neurological or pulmonary comorbidities (35, 39). Conversely, the latter comorbidities were not shown to be associated with a higher risk of adrenal crises according to the new study by Quinkler et al. (38). However, they did have more infections associated with adrenal crisis occurrence, confirming their immunocompromised state and inability to defend against viral infections owing to a natural killer cell dysfunction (23, 38). Eight % of annual cases of adrenal crises will need in hospital treatment and admission rates were highest in patients aged more than 60 years old (41). In children, adrenal crisis frequency was reported to be 3.4/100 patient-years and children diagnosed with salt wasting congenital adrenal hyperplasia (CAH), adrenal hypoplasia congenital (AHC) and AAI were at higher risk (42). Compared to the general population, patients with AI often complain of reduced quality of life and work capacity with more sick day leaves potentially explained by the inability of present treatment modalities to replicate circadian rhythm of cortisol production (6, 13). Another significant issue is the impact of PAI on fertility in women of reproductive age. Few cohort studies have shown that pregnancy rate decreases after diagnosis of PAI even in the absence of associated POI, and a higher risk of cesarean delivery, impaired fetal growth, preterm birth and low birth weight is found (43). Particularly, a German study including 39 women with CAH secondary to 21-hydroxylase deficiency and 54 with AAI, reported reduced fertility only in classic CAH and APS type 2, based on answers from a self-reporting questionnaire (44). This suggests that a more severe course of disease and/or the presence of other comorbid autoimmune disorders largely impact fertility outcomes. There are no clear explanations for the underlying mechanisms of reduced fertility in women with AAI, in the absence of POI. Perhaps their reduced quality of life and overall dissatisfaction with current treatment modalities may play a significant role in hindering successful pregnancies.

## Etiologies

The most common cause of PAI in the adult population is autoimmune destruction of the adrenal cortex (1). Other potential causes include infections, infiltrative diseases, adrenal hemorrhage, surgery, and drugs (1, 6). They are summarized in **Table 1**. AAI remains a diagnosis of exclusion and in the absence of positive antibodies directed against 21-hydroxylase, other diagnoses should be explored; an abdominal computed tomography should be performed. In the event of enlarged adrenal glands, differential diagnoses include an active tuberculous infection, systemic fungal infections in immunocompromised patients, metastases and lymphoma (1). In young men with negative antibodies to 21-hydroxylase, adrenoleukodystrophy should be suspected, even in the absence of neurological symptoms (1, 6). Adrenoleukodystrophy is an X-linked recessive disorder, hence affecting only boys, characterized by defects in *ABCD1* gene causing elevated serum very long chain fatty acids. The clinical spectrum of adrenoleukodystrophy includes cerebral manifestations that may manifest in childhood such as behavior changes, school difficulties, cognitive deficits up to dementia, psychoses and loss of vision and speech. It may be associated with adrenomyeloneuropathy presenting later in middle age with progressive lower body stiffness and weakness (45). The diagnosis of adrenoleukodystrophy should be confirmed by molecular genetic testing (46). In children, however, CAH represents 83% of PAI diagnoses compared to 9.7, 6.1 and 1.2% for AHC, autoimmune AI and adrenoleukodystrophy, respectively (42). 97.2% of CAH were secondary to 21-hydroxylase deficiency in a Chinese pediatric cohort followed up for 29 years (47). A detailed discussion of these diagnoses is outside the scope of this review and will be discussed elsewhere.

**Table 1:** Etiologies of primary adrenal insufficiency in adults (1, 5, 6, 45).

*Etiology*	*Associated clinical/biological and radiological features*
	
**AUTOIMMUNE ADRENALITIS**	Bilateral adrenal atrophy on computed tomography.
** ISOLATED**	Polygenic- positive 21-hydroxylase antibodies ± adrenal cortex antibodies/17-hydroxylase antibodies/steroid side chain cleavage enzyme autoantibodies- other AI diseases associated.
** APS TYPE 1**	Autosomal recessive AIRE mutation- positive interferon antibodies- chronic mucocutaneous candidiasis, hypoparathyroidism, ectodermal dystrophy.
** APS TYPE 2**	Polygenic- Type 1 diabetes and autoimmune thyroid disease
**INFECTIONS**	
** ACTIVE TUBERCULOUS INFECTION**	Enlarged adrenal glands on computed tomography, calcifications may be seen.
** SYSTEMIC FUNGAL INFECTIONS**	
** WATERHOUSE-FRIDERICHSEN SYNDROME**	Altered mental state, hypotension, fever, acute adrenal crisis.
** SEPTIC CHOC**	
** OPPORTUNISTIC INFECTIONS IN IMMUNOCOMPROMISED**	
**INFILTRATIVE DISEASES**	Enlarged adrenal glands on computed tomography.
** METASTASES FROM LUNG, BREAST, OR KIDNEY CARCINOMAS**	Known primary cancer.
** SARCOIDOSIS**	Other signs specific to infiltrative disease (mediastinal lymph nodes, chronic kidney disease, hypoparathyroidism, diabetes mellitus…)
** AMYLOIDOSIS**	
** HEMOCHROMATOSIS**	Hypophysitis may be associated with infiltrative diseases.
**ADRENAL HEMORRHAGE**	Sudden pain accompanied with acute adrenal crisis.
**COAGULATION DISORDERS**	Adrenal hemorrhage on computed tomography.
**ANTICOAGULANT TREATMENT**	Warfarin.
**ANTIPHOSPHOLIPID SYNDROME**	Positive cardiolipin antibodies, lupus anticoagulant and anti-beta-2 glycoprotein 1.
**BILATERAL ADRENALECTOMY**	
**PRIMARY ADRENAL LYMPHOMA**	Enlarged adrenal glands on computed tomography.
**DRUGS**	
** INCREASE CORTISOL METABOLISM**	Induction of P450-cytochrome enzymes, CYP3A4, CYP2B1, CYP2B2: phenytoin, rifampicin, phenobarbital
** IMPAIRED STEROIDOGENESIS**	Ketoconazole, fluconazole, mitotane, metyrapone, etomidate, aminoglutethimide, trilostane. Abiraterone acetate.
** ANTAGONIZE GLUCOCORTICOID ACTION ON PERIPHERAL TISSUES**	Mifepristone
** ADRENOLYTIC**	Mitotane
** TRIGGER AUTOIMMUNE REACTION**	Nivolumab and pembrolizumab
**ADRENOLEUKODYSTROPHY**	Young men
	X-linked recessive
	Defects in *ABCD1* gene
	Negative antibodies to 21-hydroxylase
	Elevated serum very long chain fatty acids
	Progressive neurological deficit, hypogonadism

## Pathogenesis of Autoimmune AI

AAI is an autoimmune destructive process affecting all three zones of the adrenal cortex in which lymphocytes infiltrate the adrenal parenchyma leading to adrenal fibrosis and atrophy. It is a slow process and AI may not be clinically relevant until most of adrenocortical cells are destroyed (48). Antibodies directed against 21-hydroxylase are specific to AAI and are rarely seen in the general population; hence their presence alongside positive adrenal cortex antibodies allow to accurately diagnose AAI in 99% of cases (19, 49, 50). The presence of antibodies targeting interferon-α2 and interferon-ω should prompt genetic testing for APS type 1 (19, 49). While the detection of anti 21-hydroxylase antibodies is diagnostic of AAI, they have no direct role in pathogenesis and are only biologic markers of autoimmunity (48, 49). In fact, cytotoxic T-cells auto-reactive to steroidogenic enzymes, in particular 21-hydroxylase, infiltrate the adrenal cortex in response to proinflammatory chemokines, CXCL9 and CXCL10, released intrinsically by adrenocortical cells. Hence, adrenocortical cells contribute to their own destruction in the presence of both, a genetic predisposition and environmental triggers (48). Possible triggers are thought to be caused by local viral infections with increased tropism to adrenocortical cells, such as herpes simplex virus 1, cytomegalovirus and adenovirus, as well as interferon alfa treatments and the relatively new checkpoint inhibitors targeting CTLA-4, programmed cell death protein 1 (PD-1), and PD-1 ligand (PD-L1), used in melanoma and lung cancer treatment (48). Genetic variants implicated in AAI development include the autosomal recessive mutation in AIRE gene commonly responsible for APS type 1 (50) but was also implicated in AAI independently of APS type 1 in a recent genome wide association study (51), which reported 2 new alterations in *AIRE*, the strongest one being p.R471C. It also described nine independent risk loci implicated in central immunological tolerance (51), in particular PTPN22, CTLA4, and BACH2 loci whose role in pathogenesis is already established (50, 51) and SH2B3 and SIGLEC5 (51). HLA class II genes also play a central role in predisposition to isolated AAI or APS type 2/4. While HLA DRB1-0301 and DRB1-040 are associated with AAI, it seems that DRB1-0403 is protective against development of Addison’s disease (50). Other polymorphisms worth mentioning include the MHC class I chain-related gene A (MICA) allele 5.1, the CIITA (MHC class II transactivator), the master regulator of MHC class II expression, STAT4, PD-L1, and the vitamin D receptor (50). All susceptibility loci described to date are in genes involved in adaptive or innate immunity (52), particularly affecting regulatory T-cell function leading to local intra-adrenal self-reactive cytotoxic T-cells (53). However, novel therapies targeting the cellular immune response or specific genes implicated in AAI pathogenesis have yet to emerge.

## Establishing the Diagnosis

**Figure 2:**
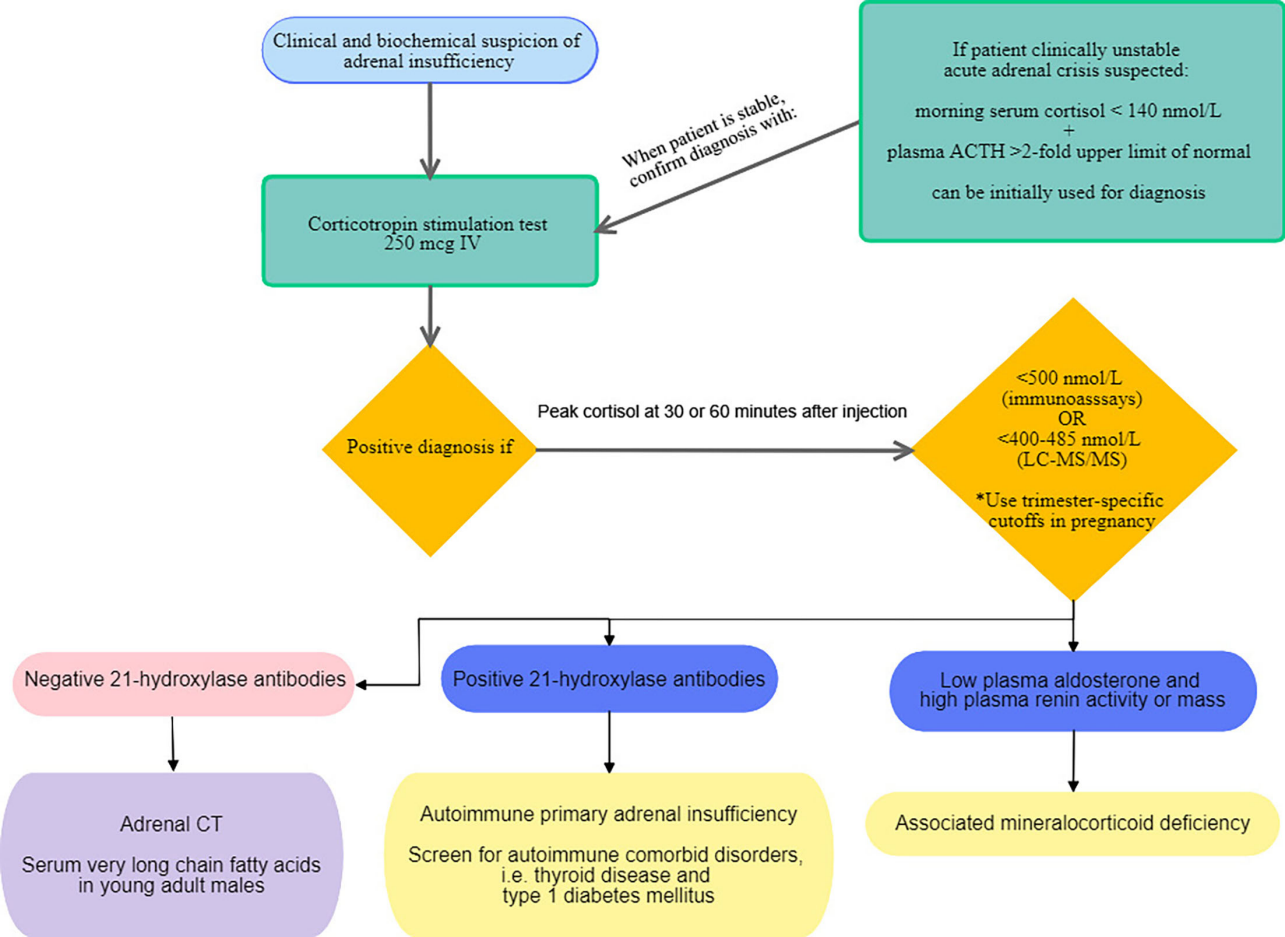
Proposed diagnostic approach for primary adrenal insufficiency in adults.

The Endocrine Society Clinical Practice Guideline published in 2016 recommend confirming the diagnosis of PAI with a corticotropin stimulation test to assess adrenocortical function (3). An algorithm depicting the proposed diagnostic approach in PAI in adults is shown in **Figure 2**. In PAI, adrenal glands are unresponsive to corticotropin stimulation because zona fasciculata is already maximally stimulated by elevated endogenous ACTH (54) and because adrenal cortex is replaced by fibrous tissue (48). In cases when confirmatory test is not possible, a morning serum cortisol of less than 140 nmol/L paired with a morning plasma ACTH above 2-fold the upper limit of normal is consistent with PAI. Measuring plasma renin and aldosterone is also recommended to document mineralocorticoid deficiency (3). There are two types of synthetic corticotropin analogs (ACTH 1-24) that can be used in the corticotropin stimulation test: cosyntropin (Cortrosyn, Amphastar Pharmaceuticals. Inc) and tetracosactrin (Synacthen, Novartis Pharma, Switzerland) and since they both exist in 250 µg formulations, the standard dose test is more practical and is recommended by the Endocrine Society Clinical Practice guideline (3). Diagnostic cutoff values of serum cortisol depend on the assay used: in most immunoassays, 500 nmol/L is often used as the cutoff to establish diagnosis (6). However, newer monoclonal immunoassays such as the Elecsys^®^Cortisol II from Roche Diagnostics, have lower cross reactivity with other steroids, thus giving values that are 20-30% lower than those with older assays (55, 56). Two different studies suggested new cutoff thresholds when using the Elecsys^®^Cortisol II: 374 nmol/L (55) and 403 nmol/L at 30 minutes (56). Furthermore, because liquid chromatography tandem mass spectrometry (LC-MS/MS) measures cortisol more accurately than immunoassays, cutoff values were also redefined to be lower, respectively 400-412 and 485 nmol/L, at 30 and 60 minutes following corticotropin analog injection (56, 57). This would potentially reduce overdiagnosis of AI. Additionally, a baseline cortisol less than 55 nmol/L successfully predicted abnormal response to stimulation test (56). A recent retrospective study conducted on 370 patients in Spain (58), confirmed need for sex-specific and assay-specific cutoff values when interpreting corticotropin test to reduce false positives and increase specificity. It also allowed for a better diagnostic agreement between sampling times at 30 and 60 minutes compared to using general cutoff values. Sampling at 30 minutes following injection of corticotropin analog accurately diagnosed 95% of PAI. However, this was not true for central AI, where sampling at 60 minutes showed better diagnostic accuracy (58). Corticotropin stimulation test can be done anytime of the day because in PAI response to ACTH is independent of circadian rhythm, but morning testing might be more accurate to avoid overdiagnosis in healthy individuals (59, 60). A metanalysis including 13 studies showed that the low dose corticotropin test (1 µg) had better accuracy for diagnosing central AI compared to the standard dose (250 µg) (61). Even so, the low dose is less practical because it necessitates dilution from supplied ampules of 250 µg and is subject to human and technical errors (3, 61). Also, samples are best withdrawn 20 to 30 minutes following injection in order to avoid false positives (59, 61). Neither corticotropin stimulation test nor metyrapone test often used to diagnose central AI, can replace insulin tolerance test considered to be the gold standard for evaluation of hypothalamic pituitary axis. A study by Giordano et al. including 31 patients with central AI, failed to demonstrate superiority of either low dose or standard dose, when both achieved same diagnostic accuracy (62). Another potential role for low dose corticotropin test would be in establishing diagnosis of latent AAI. In fact, it was found abnormal in 88.4% of 33 patients with abnormal cortisol response compared to 66.6% for the standard dose, suggesting that the standard dose might miss some cases of latent PAI (63). More studies are needed to elucidate the diagnostic role of low dose corticotropin testing. Moreover, several factors may affect interpretation of cortisol response to stimulation tests. In particular, cortisol binding globulin (CBG) which binds the majority of circulating cortisol, leaving only 5-10% of plasma cortisol free (1, 6), can be responsible for pitfalls in diagnosis. Because assays measure total serum cortisol and not free cortisol, conditions increasing CBG such as pregnancy, oral contraceptive pills and mitotane, lead to normal cortisol values, falsely reassuring clinicians (6, 64). Hence, trimester-specific cutoff values were suggested to eliminate false negatives: 700, 800 and 900 nmol/L, respectively in first, second and third trimester (65). In situations where CBG is reduced (sepsis, cirrhosis, nephrotic syndrome, hyperthyroidism and SERPINA6 gene polymorphisms), low cortisol values must be interpreted with caution (6, 66). In such patients, measurement of salivary cortisol has the benefit of being a direct, noninvasive measurement of free cortisol and correlates well with circadian variations of serum cortisol (67). While it is more often used in the diagnosis of hypercortisolism, a recent study demonstrated an added benefit of measuring salivary cortisol in response to corticotropin stimulation in particular in patients taking oral estrogens and in cases of indeterminate serum cortisol at 60 minutes, defined as values between 500 and 599 nmol/L (68). The diagnostic cutoff used in this study was 26 nmol/L (68). Another emerging noninvasive diagnostic test is the measurement of salivary cortisol or cortisone at 60 minutes following administration of 500 µg nasal tetracosactide with mucoadhesive chitosan. This generated the same 60-minute plasma cortisol response as seen with 250 µg intravenous tetracosactide and slightly lower levels of salivary cortisol and cortisone with the nasal formulation (69). Granted, this noninvasive diagnostic method is safe and convenient for patients, it requires additional studies before wide application can be recommended. Finally, measurement of anti 21-hydroxylase antibodies is necessary to establish autoimmune etiology. Commercially available assays include immunofluorescence and autoantibody assays. However, clinicians should keep in mind that they are not standardized and variations in between assays exist (3, 6). When AAI is diagnosed, screening for other comorbid autoimmune diseases should be undertaken; in particular autoimmune thyroid disease (70), type 1 diabetes (3) and POI, especially when steroid side chain cleavage enzyme autoantibodies are detected (29).

## Management of Chronic and Acute PAI

**Figure 3:**
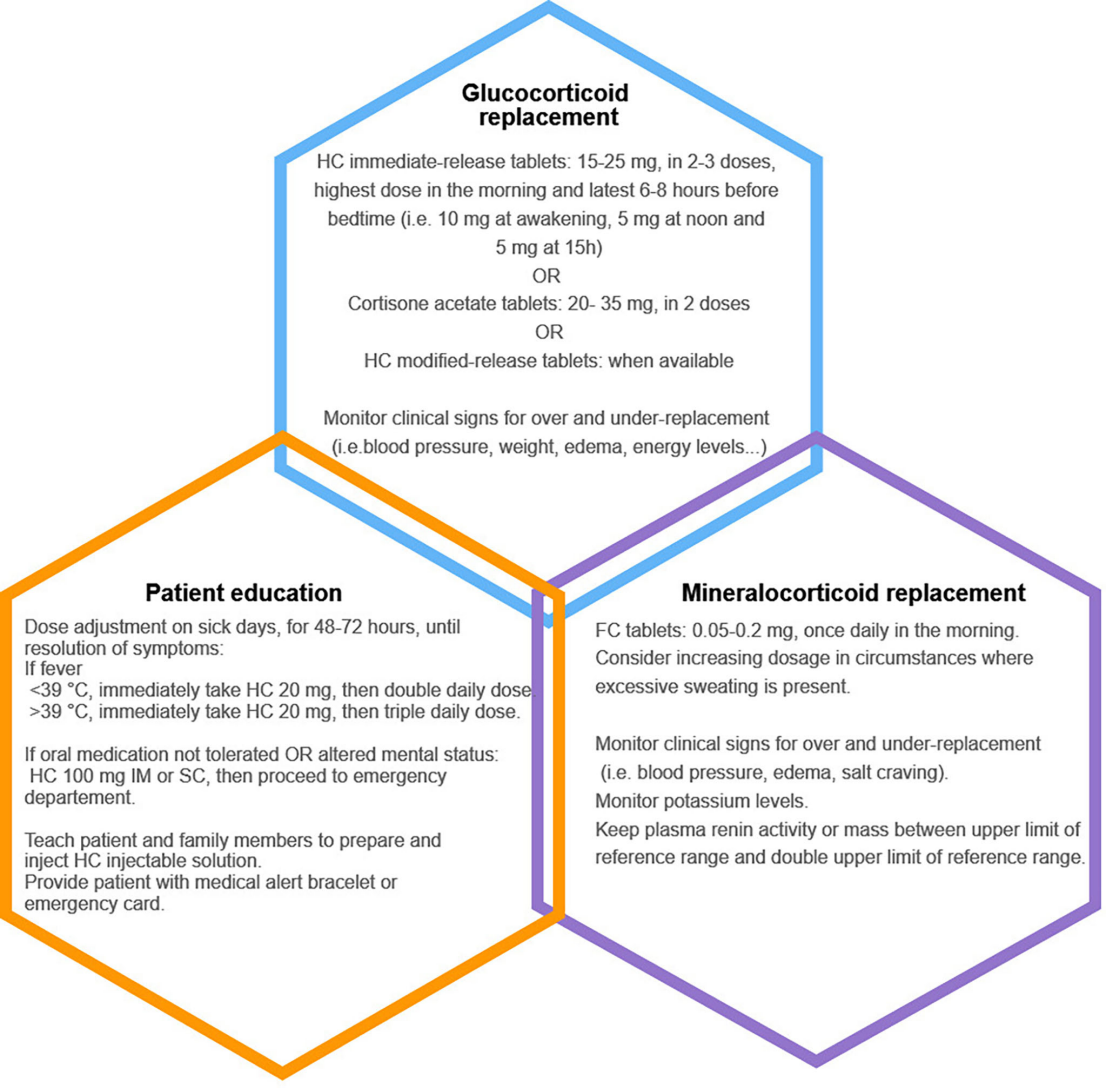
Summary of the three pillars of management in adults with primary adrenal insufficiency. HC, hydrocortisone; FC, fludrocortisone; IM, intramuscularly; SC, subcutaneously.

Cortisol release follows a circadian and ultradian rhythm. It peaks early in the morning then gradually declines to reach nadir around midnight (6). Pulsatile release of cortisol every 60-90 minutes seems to be intrinsically related to interactions between the pituitary and the adrenal glands and might be independent of supra-pituitary influences (71). The Endocrine Society Clinical Practice Guideline recommends treating all patients with PAI with glucocorticoids and mineralocorticoids when aldosterone deficiency is confirmed (**Figure 3**). The authors agreed that hydrocortisone or cortisone acetate, given in two or three divided oral doses, should be the preferred therapeutic choices (3). Total daily dose should be the equivalent of 15-25 mg of hydrocortisone for adults, with the highest dose given in the morning, in an attempt to replicate the physiologic circadian rhythm (3). However, Caetano et al. recently showed that the daily hydrocortisone dose sufficient to substitute for glucocorticoid deficiency, without signs of under replacement, in 25 adults with AI, was significantly less than that recommended by the Endocrine Society Clinical Practice Guideline (72). The mean replacement dose reported in their study was 7.6 ± 3.5 mg/m2, reflecting daily endogenous cortisol production (72). A recent systematic review of 47 studies reported that although prednisolone therapy increased risk of dyslipidemia and cardiovascular disease, it was as safe and efficacious as hydrocortisone (73). It also suggested that lower doses of hydrocortisone (less than 20 mg/day) had better clinical outcomes, and failed to conclusively demonstrate an added benefit of modified release hydrocortisone or continuous subcutaneous hydrocortisone infusion using insulin pumps (73). Current available regimens fail to replicate both circadian and ultradian rhythmicity of cortisol, potentially explaining the persistently low quality of life that patients with AI often complain about. In fact, non-pulsatile cortisol secretion was found to be associated with poor quality of sleep, poor working memory performance and mood disorders (74, 75). An in-depth discussion of novel forms of glucocorticoid substitution and their role in better mimicking physiologic cortisol secretion will be covered in other chapters of this special topic.

Overreplacement of glucocorticoids predisposes to elevated blood pressure, diabetes, osteoporosis and obesity, and thus should be avoided (6). Monitoring of glucocorticoid therapy is based solely on clinical assessment using signs and symptoms of over and under replacement such as body weight, blood pressure, energy levels, hyperpigmentation and bone mineral density (3). Use of ACTH measurement is especially unreliable and will most definitely lead to overreplacement owing to the disrupted negative feedback of cortisol on ACTH (3). Although hair cortisol concentrations were found to be useful in identifying children overtreated with hydrocortisone, it requires a 1 cm thick hair sample and the technique is not yet widely available (76). Also, gene expression emerged recently as a potential tool for monitoring hydrocortisone therapy. In particular, expression of DSIPI, DDIT4 and FKBP5 increased 2 hours after hydrocortisone infusion and correlated well with normal serum cortisol levels (77).

Mineralocorticoid deficiency is treated with fludrocortisone, approximately 50-200 mcg in adults, in one single morning dose, and ad libitum salt consumption (3). Higher doses are often needed in specific circumstances such as in children because of early mineralocorticoid resistance, in athletic people and in very hot climates because of salt wasting due to excessive perspiration (3, 6). Clinicians should look for signs of overreplacement (hypertension, peripheral edema, hypokalemia) and those of under-replacement (orthostatic hypotension, salt craving and hyperkalemia). Adequate mineralocorticoid replacement can also be determined according to plasma renin concentration, which should be kept in the upper reference range (3).

Until this day, no evidence exists to support dehydroepiandrosterone (DHEA) replacement therapy in all patients with PAI. A trial therapy of 25-50 mg of DHEA for 6 months can be initiated in premenopausal women complaining of reduced libido and depressive moods despite adequate glucocorticoid and mineralocorticoid therapy (3, 6). Unfortunately, well standardized and reliable DHEA formulations are lacking in many countries.

Patient education on dose adaptation and sick day rules is an essential part of management (**Figure 3**). Because all patients with PAI are at risk of adrenal crisis, physicians should insist on increasing patient awareness to their disease by implementing treatment strategies, participating in group sessions, and prescribing at home hydrocortisone injection kits in case of emergencies (78). Hydrocortisone dose adjustments are required during sick days: patients should double or triple their daily oral dose for 48-72 hours until full recovery. If oral medication cannot be tolerated or absorbed, 100 mg of parenteral hydrocortisone should be promptly administered before seeking medical care (3). Subcutaneous hydrocortisone injection is an alternative in patients who cannot self-inject hydrocortisone intramuscularly. It was shown that cortisol increased rapidly following subcutaneous hydrocortisone with a delay of only 11 minutes when compared to intramuscular hydrocortisone (79). A medical alert bracelet or an emergency card can help health care providers identify patients with PAI requiring lifesaving hydrocortisone administration in emergent situations (78). During adrenal crises, it is recommended to administer 100 mg of intravenous hydrocortisone followed by either continuous infusion of 200 mg of hydrocortisone per 24 hours or 50 mg intravenously every 6 hours alongside adequate isotonic saline infusion. Tapering of hydrocortisone can begin 24-48 hours following adrenal crisis, when patients can tolerate oral medication. Fludrocortisone can be re-introduced when hydrocortisone dose is less than 50 mg/day (3, 6). Stress dosing is also needed before dental and minor surgeries (25-75 mg/24h of hydrocortisone, depending on type of procedure). Major surgeries, trauma and delivery require same dosing regimen as in adrenal crises management (3). Management of PAI in special populations including children, patients with CAH, pregnant women and endurance athletes is not discussed in this review.

## Residual Glucocorticoid Function and Future Perspectives

Adrenocortical plasticity is a well-established concept defined by the ability of subcapsular adrenocortical stem cells to proliferate, then migrate into the three zones of the adrenal cortex where zone-specific differentiation occurs, and cells acquire steroidogenic function; all under the influence of ACTH (54). Adrenal mass is also influenced by ACTH: when deficient, adrenal atrophy and hypofunction develop, whereas when increased, adrenal hyperplasia and hyperfunction occur (54). Adrenocortical stem cells may hold the key to understanding the mechanisms of a new emerging concept in autoimmune adrenalitis; that of residual glucocorticoid function. Indeed, residual glucocorticoid production was described in 30-50% of patients years after AAI was diagnosed (80, 81), more commonly in men and in those with a more recent diagnosis. In contrast, residual mineralocorticoid production was only found in 13.5% of patients (80). It was hypothesized that the lack of expression by adrenocortical stem cells of 21-hydroxylase and other enzymes implicated in steroidogenesis, may protect adrenal cortex from complete autoimmune destruction by retaining the possibility of repopulation by intact stem cells (54). Also, turnover of adrenocortical stem cells differs according to sex in rodents and could possibly explain why endogenous residual function is more commonly observed in men (54). Elevated ACTH in PAI may also contribute to the stimulation of proliferation and differentiation of adrenocortical stem cells. However, once hydrocortisone replacement therapy is introduced, endogenous cortisol production declines alongside the ACTH decline and it is not known whether this is responsible for a more rapid loss of residual function or it is but the natural history of autoimmune adrenalitis that is responsible for the loss of function over time (82). Glucocorticoid precursors such as 11-deoxycortisol, 11-deoxycorticosterone and corticosterone, are potential biomarkers of residual endogenous adrenal function because their concentrations correlated well with serum cortisol (81, 82). Although, some patients retained residual adrenal function, they were not protected from increased risk of adrenal crisis and quality of life was still significantly altered (80). Spontaneous recovery of endogenous adrenal function in AAI is rarely described in the literature, 7-16 years following diagnosis, with most cases being partial recovery (83–85). Recent studies suggested that treatment with immunomodulators could be a step forward to reverse autoimmune destruction and allow for regeneration of adrenal cortex. Two of 13 patients who were given subcutaneous tetracosactide for a period of 20 weeks had urine glucocorticoid metabolite in the median range of healthy individuals and peak serum cortisol concentrations above 400 nmol/L, allowing for cessation of glucocorticoid therapy (86). A more recent study evaluated the effect of dual therapy with rituximab and depot tetracosactide in 13 patients with AAI (87). Although dual therapy did not allow full recovery of adrenal function, as defined in this study by peak cortisol > 550 nmol/L at week 48 of treatment, it showed that endogenous cortisol production, quantified by urine metabolites, was increased in 62% of patients (87). Much remains to be explored in regenerative therapies and their role in recovering adrenal function. More studies are needed to allow for a better treatment approach that will mimic physiologic production of cortisol in order to ameliorate quality of life and reduce the morbidity related to PAI.

## Conclusion

AAI remains a life-threatening condition if not recognized early. Despite medical advances, adrenal crises still occur and quality of life of patients is largely impacted. Attention should be especially given to patients with latent AI, at early stages of adrenal destruction, to prevent adrenal crises from developing and going unnoticed. Impending novel therapies are being explored to determine the best approach to utilize residual adrenal cortisol production and to regenerate adrenocortical cells destroyed by the autoimmune process. Until this is a validated practice, the only effective treatment remains adequate glucocorticoid and mineralocorticoid replacement and patient and physician education.

## Author Contributions

NY, IB, and AL contributed to conception and design of this review. NY wrote the first draft of the manuscript. IB and AL wrote sections of the manuscript and revised the final draft. All authors contributed to the article and approved the submitted version.

## Conflict of Interest

The authors declare that the research was conducted in the absence of any commercial or financial relationships that could be construed as a potential conflict of interest.

## Publisher’s Note

All claims expressed in this article are solely those of the authors and do not necessarily represent those of their affiliated organizations, or those of the publisher, the editors and the reviewers. Any product that may be evaluated in this article, or claim that may be made by its manufacturer, is not guaranteed or endorsed by the publisher.
